# The specifics of the Galois field *GF*(257) and its use for digital signal processing

**DOI:** 10.1038/s41598-024-66332-2

**Published:** 2024-07-04

**Authors:** Akhat Bakirov, Dinara Matrassulova, Yelizaveta Vitulyova, Dina Shaltykova, Ibragim Suleimenov

**Affiliations:** 1 Al-Farabi Kazakh National University, Almaty, Kazakhstan; 2 National Engineering Academy of the Republic of Kazakhstan, Almaty, Kazakhstan

**Keywords:** Galois fields, Mersenne numbers, Quasi-cyclic permutations, Digital logarithm, Modulo multiplication algorithm, UAV flight computers, Applied mathematics, Computational science, Information technology

## Abstract

An algorithm of digital logarithm calculation for the Galois field $GF(257)$ is proposed. It is shown that this field is coupled with one of the most important existing standards that uses a digital representation of the signal through 256 levels. It is shown that for this case it is advisable to use the specifics of quasi-Mersenne prime numbers, representable in the form ${p=2}^{n}+1$, which includes the number 257. For fields $GF({2}^{n}+1)$, an alternating encoding can be used, in which non-zero elements of the field are displayed through binary characters corresponding to the numbers + 1 and − 1. In such an encoding, multiplying a field element by 2 is reduced to a quasi-cyclic permutation of binary symbols (the permuted symbol changes sign). Proposed approach makes it possible to significantly simplify the design of computing devices for calculation of digital logarithm and multiplication of numbers modulo 257. A concrete scheme of a device for digital logarithm calculation in this field is presented. It is also shown that this circuit can be equipped with a universal adder modulo an arbitrary number, which makes it possible to implement any operations in the field under consideration. It is shown that proposed digital algorithm can also be used to reduce 256-valued logic operations to algebraic form. It is shown that the proposed approach is of significant interest for the development of UAV on-board computers operating as part of a group.

## Introduction

Binary and non-binary Galois fields are increasingly used in information technology. Mostly such algebraic structures are used to develop information security algorithms^1–3^, i.e. in the area that obviously operates with alphanumeric symbols that can be put in correspondence with the elements of one or another discrete set studied by abstract algebra. Very significant results have been obtained in this area, using, among other things, Fourier-Galois transformations^4–6^. Analysis of current literature in this area, in particular, such reports as^7–9^, allows us to assert that coding theory (including the theory of error-correction codes^10^) has already in many ways become a part of applied abstract algebra. For developing information security algorithms, other nontrivial algebraic structures are used, in particular, finite algebraic rings^11–13^.

It is appropriate to emphasize that the needs of practice, in particular those related to the calculation of Fourier-Galois transforms^14^, force one to turn to the use of non-trivial code systems^15^ too.

However, the use of finite algebraic structures is of interest not only from the point of view of cryptography (more widely—information security systems). In particular, the creation of groups of unmanned aerial vehicles (UAVs) operating as a group and controlled by a single operator is currently attracting more and more attention of researchers^16,17^. Creating algorithms for controlling such groups is a non-trivial task^18–20^. Any such algorithms are based on the fact that the onboard computer of an individual UAV processes not only the commands received from the operator, but also the information received from the other UAVs in the group. All this information must be converted into executable commands to be fed to the actuating units of a particular UAV. It is significant that fuzzy logic is nowadays increasingly used to solve such a problem^21,22^. The number of variables corresponding to such logic is known to be finite, which was clearly demonstrated in^23^ using the example of the rhumb rose. Moreover, as shown in the cited work, the values of fuzzy logic variables can be put in correspondence with the values of variables of multivalued logic, and such a comparison for practical purposes does not necessarily have to be mutually unambiguous. In particular, "empty" commands can be introduced into consideration. This allows us to use the most convenient variants of multivalued logics, in particular, those of them, the set of values of variables of which can be mutually unambiguously matched to the set of elements of the Galois field $GF\left({\text{p}}^{\text{n}}\right)$.

Moreover, any information exchanged between the elements of the UAV group, both among themselves and with the operator, is usually represented in a digital form, and one that meets existing standards. One of the most common is the 256-signal level standard. Therefore, even if we do not consider the application of fuzzy logic to the control of UAV groups, this issue can be solved in terms of multi-valued logic, and the relevant issue is the choice of logic that meets the existing standards.

This example allows us to take a somewhat broader view at the applied use of abstract algebra tools^23^. Namely, in the natural science tradition, physical phenomena are, as a rule, described by functions that take real or complex values. However, as emphasized in^24,25^, this is nothing more than a matter of agreement. A function that takes values in a particular set is nothing more than a model of a real process, for example, a signal^24,25^. From a general methodological point of view, there is a mapping of a real physical process (for example, signal generation) onto a certain mathematical object, the choice of which is ultimately determined by issues of convenience and efficiency of use.

In particular, for a digital signal varying in a finite range of amplitudes, a function that takes values in Galois fields^24,25^ or algebraic rings^26,27^ can be used as a signal model. The number of discrete levels that fit into a finite range of amplitudes is also finite. Consequently, a function that takes values in any algebraic structure with a finite number of elements can be used for description of processes of this kind.

This fully correlates with the above statement: for a number of applications (including the development of algorithms for controlling groups of UAVs) it is acceptable to operate with elements of finite algebraic structures.

We emphasize that the elements of the Galois field can be assigned to the values of a multivalued logic variable. Moreover, as shown in^23,28^, it is possible to reduce any logical operations to algebraic form. We emphasize that traditionally functions that depend on a logical variable are presented in tabular form^29,30^. The results obtained in^23,28^ allow one to convert such tables to explicit algebraic expressions. Such expressions can be used, for example, as a basis for on-board UAV calculators acting as part of a group due to the finite number of executable commands and the possibility of representing the information on the basis of which they are generated, in terms of fuzzy logic.

One of the most commonly used standards divided the amplitude range into 256 levels^31^. This standard corresponds to the Galois field $GF\left({2}^{8}\right)$, i.e. each of the signal levels can be associated with an element of this field.

Consequently, it is permissible to consider the problem of reducing calculations corresponding to such a number of discrete levels to calculations in Galois field. It is appropriate to note that there are reports devoted to the development of electronic circuits that perform modulo addition and multiplication operations in current literature, for example,^32,33^. Such operations correspond to operations in the simplest Galois fields $GF\left(p\right)$, where $p$ is a prime number. The proposed formulation of the problem fully meets this trend.

From the point of view of applied purposes, different Galois fields have different specifics^34,35^, i.e. it is not always justified to consider the question of the maximum generalization of the results obtained using a specific Galois field. In particular, the field $GF\left({2}^{4}+1\right)$, studied in^36^, is conjugate to the field GF(${2}^{4}$), which makes it possible to bring into explicit form any operations for the important special case of 16-valued logic. The fact that the number 17 is one of the quasi-Mersen primes, representable in the form $p={2}^{n}+1$, is used in^36^.

Moreover, from the point of view of solving the problem of controlling groups of UAVs (and similar ones), it is acceptable to raise the question of creating universal calculators oriented to the use of a sufficiently large number of commands. The indicator corresponding to 256 levels, obviously overlaps the existing needs (especially if we take into account that the UAV course correction can correspond to a discrete rhumb rose^23^). Consequently, if we focus on this existing standard, it is possible in the future to raise the question of creating universal onboard calculators, allowing the use of different variants of fuzzy logic.

The number of levels equal to 256 is associated with one of the quasi-Mersenne numbers (257), which makes it possible to significantly simplify any computational procedures associated with carrying out calculations in the field $GF\left({2}^{8}\right)$ due to transition to calculations in the conjugate Galois field $GF\left(257\right)$.

This paper presents specific algorithms that can significantly simplify any calculations in the field $GF\left(257\right)$, and also presents specific electronic circuits that prove the effectiveness of the proposed algorithms. It is important that these algorithms, among other things, make it possible to implement calculations in the field under consideration based on a standard element base corresponding to binary logic.

The basis of these algorithms is, in particular, the operation of digital logarithm. This issue is also quite actively discussed in the literature^37–39^, and there are examples when this problem is considered in relation to a specific Galois field^40^.

In this work, it is proved that the specificity of the $GF\left(257\right)$ field makes it possible to implement a digital logarithm algorithm, which can be used to create electronic circuits, including those that perform operations in 256-valued logic, i.e. one of the most important technical standards.

## Methods: comparison of Mersen and quasi-Mersen numbers using for digital signal processing

The method used in this report is based on the use of prime numbers, which can be called quasi-Mersen numbers.

To make the comparison adequate, let us briefly consider the basic properties of Mersenne numbers, which currently find concrete practical applications^41,42^.

Such numbers can be represented in the form

1 $$p={2}^{n}-1$$

where $n$—are specifically chosen integers, the first of which are 2, 3, 5, and 7.

In relation to digital signal processing, the following property of Mersenne numbers is of interest, which is convenient to consider using the example of the field $GF\left(127\right)$ whose characteristic is the Mersenne number with $n=7$.

In binary form, any of the elements of the $GF\left(127\right)$ field can be represented as

2 $$A={a}_{6}{a}_{5}\dots {a}_{1}{a}_{0}.$$

where $${a}_{i}=\text{0,1}$$

Multiplying a given number by 2 modulo 127 is reduced to a cyclic permutation of symbols

3 $$2{a}_{6}{a}_{5}\dots {a}_{1}{a}_{0}={a}_{5}\dots {a}_{1}{a}_{0}{a}_{6}$$

Formula (3) is a consequence of the next relation

4 $$1111111\equiv 0000000\left(127\right)$$

Property (3), in particular, makes it possible to significantly simplify the algorithm for multiplying numbers modulo 127 by each other.

A similar algorithm was proposed for particular case of quasi-Mersen numbers in^29^.

Such numbers can be represented in the form

5 $$p={2}^{n}+1$$

The algorithm^36^ is based on the following properties of quasi-Mersen numbers, which can be conveniently considered using the field $GF\left(17\right)$ as an example. This field can be considered as a set of elements

6 $$GF\left(17\right)=\left\{-8,-7,\dots ,0,\dots \text{7,8}\right\}$$

since the choice of representing elements is arbitrary up to the modulo comparison operation, for example, $-8\equiv 9(17)$*.*

All non-zero elements of the field under consideration satisfy the relation

7 $${x}^{16}-1=0$$

From this relation, in particular, it follows that any element of the field under consideration can be represented in the form

8 $$x={\left(\sqrt[2]{1}\right)}^{{s}_{4}}{\left(\sqrt[4]{1}\right)}^{{s}_{3}}{\left(\sqrt[8]{1}\right)}^{{s}_{2}}{\left(\sqrt[16]{1}\right)}^{{s}_{1}}$$

where ${s}_{i}=0;1$, and the notation $\sqrt[{2}^{k}]{1}$ denotes an element having next properties:

9 $${\left[\sqrt[{2}^{k}]{1}\right]}^{{2}^{k}}=1; {\left[\sqrt[{2}^{k}]{1}\right]}^{{2}^{k-1}}\ne 1$$

Specifically, for the field $GF\left(17\right)$ such elements are equal

10 $${\left(\sqrt[16]{1}\right)}_{1}=-1$$

11 $${\left(\sqrt[16]{1}\right)}_{2}=4;-4$$

12 $${\left(\sqrt[16]{1}\right)}_{3}=2;8;-8;-2$$

13 $${\left(\sqrt[16]{1}\right)}_{4}=3;5;6;7;-7;-6;-5;-3$$

The possibility of using representation (8) follows from the general theory of Galois fields. Indeed, all powers of any primitive element $y$ of the field under consideration from 0 to 15 are different. At the same time, all these powers are roots of Eq. (5), i.e. they exhaust the elements of the field.

Let us consider the degree ${y}^{m}$, and represent the number m, where $0\le m\le 15$ in binary form

14 $$m={m}_{4}{m}_{3}{m}_{2}{m}_{1}$$

where ${m}_{i}$ are binary characters, i.e.

15 $$m={2}^{3}\cdot {m}_{4}+{2}^{2}\cdot {m}_{3}+{2}^{1}\cdot {m}_{2}+{2}^{0}\cdot {m}_{1}$$

Therefore, the degree ${y}^{m}$ can be represented in the form

16 $${y}^{m}={\left({y}^{8}\right)}^{{m}_{4}}{\left({y}^{4}\right)}^{{m}_{3}}{\left({y}^{2}\right)}^{{m}_{2}}{\left({y}^{1}\right)}^{{m}_{1}}$$

This expression may be reduced to the form (8) by using relations (10)–(13).

Starting from an expression of the form (8), one can propose an alternating encoding^36^, which is also convenient to consider using the example of the $GF\left(17\right)$ field.

Let us consider an expression for non-zero elements of $GF\left(17\right)$ field

17 $$A={2}^{3}\cdot {a}_{3}+{2}^{2}\cdot {a}_{2}+{2}^{1}\cdot {a}_{1}+{2}^{0}\cdot {a}_{0}$$

where $${a}_{i}=\pm 1$$

The result of calculations using formula (17) will certainly give an odd number. There are ${2}^{4}$ combinations of the form (17), with the maximum number being ${A}_{max}=15$ and, accordingly, ${A}_{min}=-15$.

One can see, that the number of combinations of the form (17) in the case under consideration coincides with the number of non-zero elements of the field $GF\left(17\right)$. Thus, after modulo reduction, the numbers represented in the form (17) exhaust the set of non-zero elements of the field $GF\left(17\right)$. Consequently, this representation can be used along with any other, especially if we take into account that “representatives” of the residue classes of the ring of integers modulo a prime number can be chosen in an arbitrary way.

A representation of the form (17), in which ${a}_{i}=\pm 1$ has a property similar to the property possessed by Mersenne prime numbers (3). Namely, multiplying the number written in representation (15) by 2 can be represented as the following operation

18 $$2\cdot A={2}^{3}\cdot {a}_{2}+{2}^{2}\cdot {a}_{1}+{2}^{1}\cdot {a}_{0}-{2}^{0}\cdot {a}_{3}$$

This follows from the fact that in the field under consideration ${2}^{4}\equiv -1\left(17\right)$.

Consequently, the operation of multiplication by 2 in alternating binary representation of a number is reduced to a cyclic rearrangement of binary elements with a change in the sign of the last element. We have

19 $$2\cdot A=2\cdot {a}_{2}{a}_{1}{a}_{0}{a}_{3}={a}_{2}{a}_{1}{a}_{0}\left(-{a}_{3}\right)$$

It can be seen that this property is indeed similar to the property of the fields formed using Mersenne numbers (3).

This property gives possibility to propose simple algorithm of digital logarithm operation for very important particular case $GF\left(257\right)$ too.

## Results and discussion

### Computational invariants for elements of field GF(257)

Calculation of elements ${s}_{j}$ in representation (6) and similar ones corresponds to digital logarithm operation.

With the help of algorithms (and/or digital devices) that perform such an operation, the multiplication operation can obviously be reduced to an addition operation.

In this section it is proved that the set of non-zero elements of the field GF(257) can be divided into subsets, and this division allows one to significantly simplify digital logarithm operation. Looking ahead somewhat, we note that proposed algorithms also make it possible to significantly simplify the electronic circuits that perform this operation.

The proposed algorithm is based on quantities that can be called computational invariants. The rationale for their use is given in this section.

Let us start from the identity

20 $${x}^{256}-1={\left({x}^{16}\right)}^{16}-1,$$

The right side of relation (20) emphasizes the following fact. To represent an arbitrary non-zero element of the field GF(257) using relation (17), 8 bits are needed. Taking into account the change of sign of the last element when multiplying by 2 (16), there are 16 elements that differ from each other by the factor ${2}^{k}$, k = 0,1,…,15 in this field.

Therefore, an arbitrary non-zero element of a given field can be represented in the form

21 $$x={\left(\sqrt[16]{2}\right)}^{\sigma }{2}^{k}$$

where $$\sigma =\text{0,1},\dots , 15$$, $$k=\text{0,1},\dots ,15$$.

We emphasize that the root $\sqrt[16]{2}$ should be chosen as equal to a primitive element, i.e. the different degrees of the root must give all elements of the field $GF\left(257\right)$.

Similarly, all non-zero elements of the field $GF\left(17\right)$ can be expressed by a formula similar to (24), obtained in^36^:

22 $$x={\left(\sqrt{2}\right)}^{\sigma }{2}^{k}$$

The adequacy of representations (21) can also be proven as follows. Let us consider an arbitrary degree of a primitive element $\sqrt[16]{2}$

23 $$z={\left(\sqrt[16]{2}\right)}^{w}$$

where $$w=\text{0,1},\dots ,256$$.

Let's represent the number *w* in standard binary encoding

24 $$w={2}^{7}{w}_{7}+{2}^{6}{w}_{6}+\dots +{2}^{0}{w}_{0}$$

Let's substitute expression (24) into formula (23). We have

25 $$z={\left({2}^{4}\right)}^{{w}_{7}}{\left({2}^{3}\right)}^{{w}_{6}}\dots {\left(\sqrt[8]{2}\right)}^{{w}_{1}}{\left(\sqrt[16]{2}\right)}^{{w}_{0}}$$

Note that formula (25) is equally valid for representing the field elements $GF\left(257\right)$ both in the form − 128, − 127 ,…,0,…, 127, 128 and in the form 0 ,…, 255, 256. These representations differ only by specific “representatives” of the corresponding classes of residues are used as $\sqrt[i]{2}$ (Tables 1 and 2).

**Table 1:** Elements $\sqrt[i]{2}$ of the field $GF\left(257\right)$ in terms of positive numbers.

$\sqrt[16]{2}$	$\sqrt[8]{2}$	$\sqrt[4]{2}$	$\sqrt[2]{2}$	${\left(\sqrt[16]{2}\right)}^{16}$
27	215	222	197	2
41	139	46	60	2
54	89	211	60	2
71	158	35	197	2
82	42	222	197	2
93	168	211	60	2
108	99	35	197	2
115	118	46	60	2
142	118	46	60	2
149	99	35	197	2
164	168	211	60	2
175	42	222	197	2
186	158	35	197	2
203	89	211	60	2
216	139	46	60	2
230	215	222	197	2

**Table 2:** Elements $\sqrt[i]{2}$ of the $GF\left(257\right)$ field in representation using both positive and negative numbers.

$\sqrt[16]{2}$	$\sqrt[8]{2}$	$\sqrt[4]{2}$	$\sqrt[2]{2}$	${\left(\sqrt[16]{2}\right)}^{16}$
27	− 42	− 35	− 60	2
41	− 118	46	60	2
54	89	− 46	60	2
71	− 99	35	− 60	2
82	42	− 35	− 60	2
93	− 89	− 46	60	2
108	99	35	− 60	2
115	118	46	60	2
− 115	118	46	60	2

There are exactly 16 primitive elements $\sqrt[16]{2}$ in the field $GF\left(257\right)$. They are listed in Table 1. In Table 2 only 9 elements are presented, since in the case of alternating representation the elements of $\sqrt[16]{2}$ fall into two groups that differ in sign. This is emphasized by the 9th line in Table 2.

Thus, formula (25) shows that, up to a permutation of the indicated type, the alternating binary representation allows us to reduce all elements of the field $GF\left(257\right)$ to sixteen elements of the form

26 $$y={\left(\sqrt[2]{2}\right)}^{{w}_{3}}{\left(\sqrt[4]{2}\right)}^{{w}_{2}}{\left(\sqrt[8]{2}\right)}^{{w}_{1}}{\left(\sqrt[16]{2}\right)}^{{w}_{0}}$$

The remaining elements of the $GF\left(257\right)$ field can be obtained from these sixteen elements by cyclic permutation with a change in the sign of the symbols in the alternating binary representation.

Further, representation the elements of the field $GF\left(257\right)$ in an alternating binary encoding allows one to form function ${Q}_{j,j-1}$.

27 $${q}_{i}={Q}_{j,j-1}=\left\{\begin{array}{c}1, { a}_{j}{a}_{j-1}=-1\\ 0, { a}_{j}{a}_{j-1}=+1\end{array}\right.$$

This function provides a count of the number of sign changes in code sequences corresponding to the alternating representation of an element of the field. In the case under consideration, this function contains 8 clock cycles.

**Figure 1:**
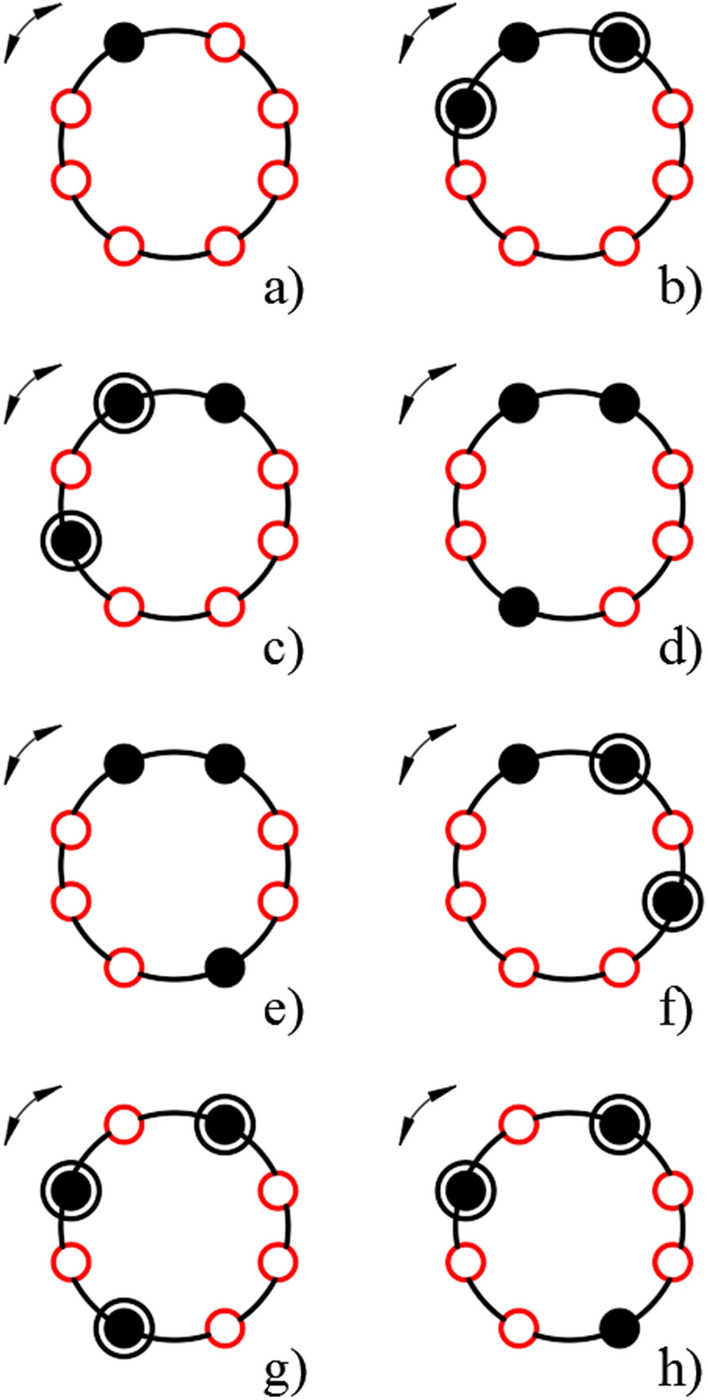
Geometric interpretation of the elements given by formula (27); black circles are vertices corresponding to values equal to 1 in formula (27), black circles with an additional black circle correspond to the case when vertices corresponding to such values are separated by one vertex.

Otherwise, we can assume that this function “takes values” at the vertices of the octagon, as shown in Fig. 1. Multiplication by powers of two in this geometric representation corresponds to the rotation of the octagon around the axis of symmetry by an angle multiple of $\frac{\pi }{4}$.

Consequently, values (27) actually correspond to well-defined geometric constructions (Fig. 1). Due to the fact that cyclic permutations with a change in sign are used, the number of non-zero values ${q}_{i}$ of function (27) can only be odd.

This geometric interpretation is illustrated by Table 3. It shows that for each specific number presented in an alternating encoding, there are certain invariants that correspond to the situations presented in Fig. 1.

**Table 3:** Invariants of sequences corresponding to alternating encoding (examples).

n	q_7_	q_6_	q_5_	q_4_	q_3_	q_2_	q_1_	q_0_	Σq_i_
− 255	0	0	0	0	0	0	0	1	1
− 253	0	0	0	0	0	0	1	0	1
− 251	0	0	0	0	0	1	1	1	3
− 249	0	0	0	0	0	1	0	0	1
− 247	0	0	0	0	1	1	0	1	3
− 245	0	0	0	0	1	1	1	0	3
− 243	0	0	0	0	1	0	1	1	3
− 241	0	0	0	0	1	0	0	0	1
− 239	0	0	0	1	1	0	0	1	3
− 237	0	0	0	1	1	0	1	0	3
− 235	0	0	0	1	1	1	1	1	5
− 233	0	0	0	1	1	1	0	0	3
− 231	0	0	0	1	0	1	0	1	3
− 229	0	0	0	1	0	1	1	0	3
− 227	0	0	0	1	0	0	1	1	3
− 225	0	0	0	1	0	0	0	0	1
− 223	0	0	1	1	0	0	0	1	3
− 221	0	0	1	1	0	0	1	0	3
− 219	0	0	1	1	0	1	1	1	5
− 217	0	0	1	1	0	1	0	0	3
− 215	0	0	1	1	1	1	0	1	5
− 213	0	0	1	1	1	1	1	0	5
− 211	0	0	1	1	1	0	1	1	5
− 209	0	0	1	1	1	0	0	0	3
− 207	0	0	1	0	1	0	0	1	3

This table presents examples of ${q}_{i}$ values for different elements of the field $GF\left(257\right)$ in alternating binary encoding, as well as invariants corresponding to the number of sign changes in the sequence (last column of Table 3).

It can be seen that the examples presented in this table clearly correlate with the geometric construction of Fig. 1. Namely, the number of sign changes in the sequences under consideration is odd, and, therefore, can be equal to 1, 3, 5 and 7. The last two cases (the invariants are 5 and 7) are reduced to the first two of those indicated by the inversion 0 ↔ 1.

Consequently, only the cases $\sum {q}_{i}=1$ and $\sum {q}_{i}=3$ can be kept in consideration. They correspond to possible placements of one and three elements on the vertices of the octagon, as shown in Fig. 1, and such placement is specified up to rotation by an angle $\frac{\pi }{4}$, i.e. placements that differ in rotation by such an angle are considered identical.

The proposed invariants make it possible to significantly simplify the operation of digital logarithm and propose a specific electronic circuit that implements this operation based on standard logic elements. It is discussed in the next section.

### The operation of digital logarithm and the electronic circuit that implements it

As follows from the materials in the previous section, the problem of calculation of digital logarithm in the field $GF\left(257\right)$ is divided into two ones.

The first one is equivalent to determination of the rotation angle of the octagon shown in Fig. 1, which actually corresponds to the definition of a power of two (when multiplied modulo 257) in representation (25).

The second one corresponds to the identification of one of the configurations presented in Fig. 1. Having determined such a circuit configuration, one can automatically determine one of the elements represented by formula (26).

Let's consider the block diagram of a device for calculation of digital logarithm in accordance with the algorithm, which follows from the above. Let's consider second-order invariants, reflecting the relative position of units at the vertices of the octagon (Fig. 1). Such invariants are, in particular, the sums ${U}_{{S}_{j}}$

28 $${U}_{{S}_{j}}=\sum {q}_{i}^{\left({s}_{j}\right)}$$

of quantities ${q}_{i}^{\left({s}_{\text{1,2}}\right)}$ given by the formulas

29 $${q}_{i}^{\left({s}_{1}\right)}={q}_{i}{q}_{i-1}$$

30 $${q}_{i}^{\left({s}_{2}\right)}={q}_{i}{q}_{i-2}$$

The identification of seven cases, except for the trivial one (Fig. 1a) is of interest. In this trivial case, the digit number directly corresponds to the non-zero value ${q}_{j}$ (27).

It can be seen that the invariant ${U}_{{S}_{1}}$ is equal to 2 for the configuration shown in Fig. 1b only. Consequently, calculation of this invariant automatically gives identification of this case. In this case, the angle of rotation of the hexagon is fixed by function (29), which in this case takes only one non-zero value.

This sum ${U}_{{S}_{1}}$ also takes a non-zero value equal to 1 for the cases of Fig. 1c–f. This corresponds to the fact that there are only two nodes in the sequence located in close proximity to each other. This invariant is equal to zero for the cases of Fig. 1g and h. Consequently, the calculation of the indicated invariant allows one to identify the case corresponded to Fig. 1b and divide the remaining options into two subsets.

Calculation of the invariant ${U}_{{S}_{2}}$ automatically selects two cases from the considered set (Fig. 1d and e). In these diagrams, there are no nodes separated from each other by only one empty vertex. Additionally, the calculation of this invariant uniquely identifies the case of Fig. 1g, for which this invariant is equal to 2. For clarity, filled vertices, separated from each other by two turns at an angle of π/4, are highlighted with additional circles.

Thus, to complete the digital logarithm operation for the field under consideration, all that remains is to ensure the difference between the cases of Fig. 1c and f, as well as between the cases of Fig. 1d and e. This difference is identified by the phase shift between functions (29) and (30), which does not depend on the presence of a factor equal to a power of 2 (which is equivalent to rotations of the octagons under consideration).

Specifically, this phase shift can be determined, for example, through the calculation of invariants built on the functions

31 $${q}_{i}^{\left({s}_{3}\right)}={q}_{i+2}{q}_{i}{q}_{i-1}$$

32 $${q}_{i}^{\left({s}_{4}\right)}={q}_{i+3}{q}_{i}{q}_{i-1}$$

To use such functions, it is already important to take into account the direction; specifically, it is assumed that the direction in Fig. 1 is counted clockwise.

It can be seen that function (31) can take a non-zero value only for the case of Fig. 1c, and function (32) is only for the case of Fig. 1d. Therefore, to identify the above cases, it is enough to count the invariants $\sum {q}_{i}^{\left({s}_{3}\right)}$ and $\sum {q}_{i}^{\left({s}_{4}\right)}$.

**Figure 2:**
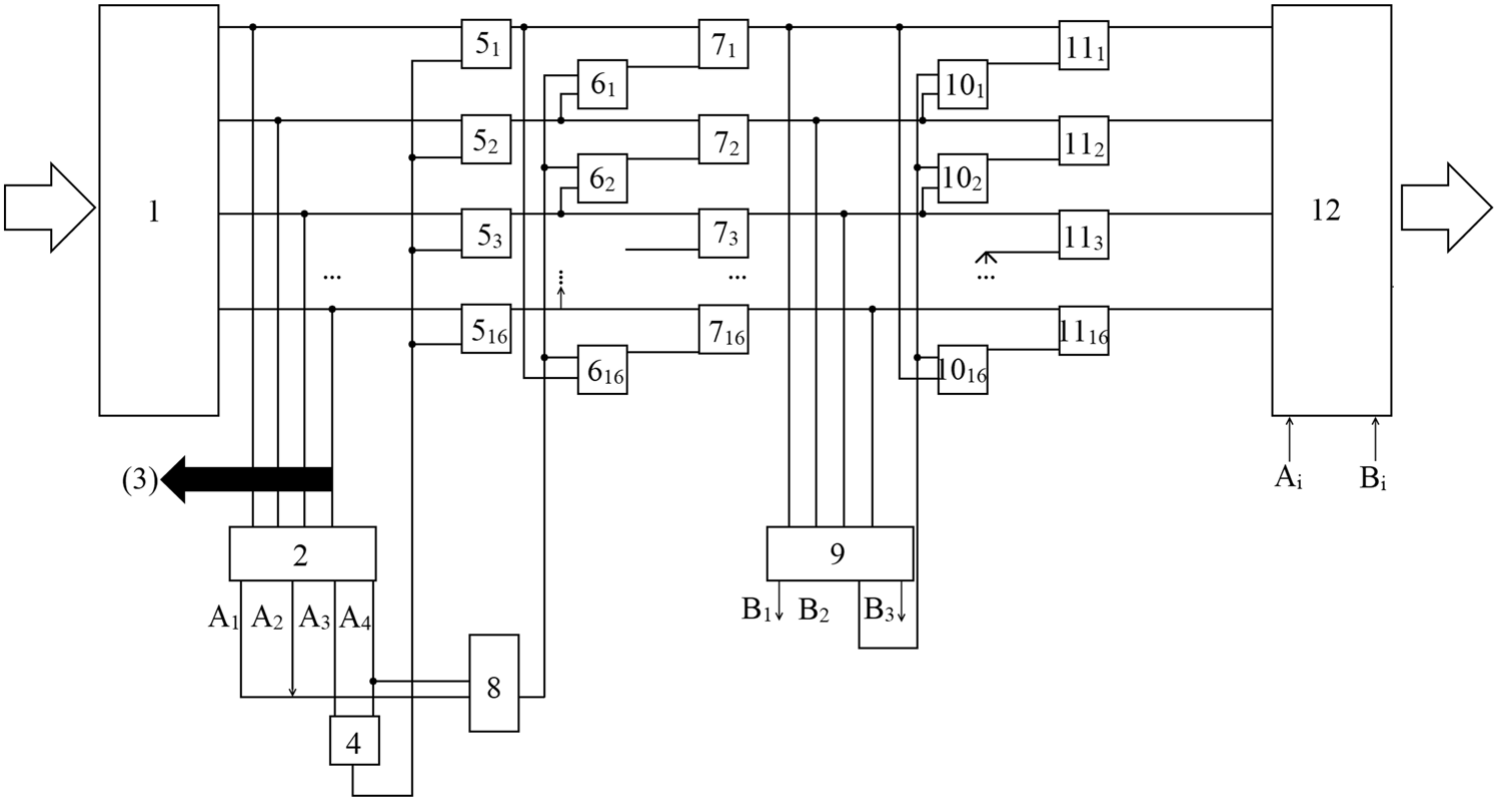
Block diagram of a device for digital logarithm in the field $GF\left(257\right)$.

As a result, we can propose the following block diagram of device for digital logarithm (Fig. 2).

A device built on this circuit works as follows.

The original binary signals (their number is 16) are sent to the converter (1), which converts them to the familiar encoding. Due to this, in particular, the set of signals generated at the output (1) can be considered cyclic.

From all 16 outputs of the converter, signals are fed to the adder (2), which counts the number of units. A logical one is formed at the output ${A}_{1}$ of the adder (2) if the number of ones is 1, i.e. the case corresponding to Fig. 1a is realized. In this case, the digital logarithm of the number is exactly equal to the number of the converter output (1) on which a signal other than 0 is generated.

A logical one is formed at the output ${A}_{2}$ of the adder (2) if the number of ones is 3, i.e. the cases corresponding to Fig. 1c-h are realized. If the number of ones is 5, then a logical one is formed at output ${A}_{3}$, and if it is 7, at output ${A}_{4}$.

Signals from outputs ${A}_{3}$ and ${A}_{4}$ are fed to the OR element (4). This signal is used to control the inverters (5_i_), which perform the inversion operation 0 ↔ 1, provided that the number of ones is 5 or 7. The inverters (5_i_) perform the addition operation modulo 2, i.e. they are EXCLUSIVE OR elements. The second inputs of the inverters (5_i_) are supplied with signals from the outputs of the converter (1).

As a result, a set of signals is formed at the outputs of the inverters (5_i_), which contain either one non-zero signal or three such signals.

This set of signals is supplied to the first stage of the first digital logarithm block, consisting of elements (6_i_) and (7_i_), as well as an OR logic element (8).

Elements (6_i_) perform a logical OR operation. One of the inputs of each of these elements is supplied with a signal from the output of the element (8), and the second is supplied with a signal from the output of the inverter (5_*i*-1_). Consequently, the signal at the output of each of the elements (6_i_) will be equal to 1 if a logical unit is formed at the output ${A}_{1}$ or output ${A}_{4}$ of the adder (2), and equal to the value of the $\left(i-1\right)$ st signal in the opposite case.

The signal from the output of each of the elements (6_*i*_)) is fed to one of the inputs of the element (7_*i*_), which performs a logical AND operation. The second input of each of these elements is fed directly from the output of the inverter (5_*i*_).

As a result, the block under consideration calculates correspondent invariant if the number of units in the set of signals at the output of the converter (1) was equal to 3 or 5 and leaves the signals generated by the set of inverters (5_i_) unchanged if the specified number was 1 or 7.

As follows from Fig. 1, this cascade already identifies sequences corresponding to Fig. 1a, as well as Fig. 1b–f in the sense that for these cases, in the set of signals generated at the outputs of elements (7_i_), only one logical unit is formed, which uniquely identifies the digital logarithm of the corresponding element of the Galois field.

To identify the remaining cases, an adder (9) is used, which counts the number of ones at the outputs of the elements (7_i_). If this number is 1, then the digital logarithm operation should be considered complete. If this number is 0, then the situations corresponding to Fig. 1g or Fig. 1h. In this case, the result of digital logarithm is generated by the second digital logarithm block, which is discussed below. Identification signals are generated at the outputs ${B}_{1}$, ${B}_{2}$ and ${B}_{3}$ of the adder (9). A logical unit is formed at these outputs if the sum of logical units at the outputs of elements (7_i_) is equal to 0, 1 and 2, respectively.

To take into account the situation corresponding to Fig. 1b, the second stage of the first digital logarithm block is used, which is designed similarly to the first. It consists of elements (10_i_) and (11_i_). Elements (10_i_) perform a logical OR operation, and elements (11_i_) perform an AND operation. The first inputs of each of the elements (10_i_) are supplied with signals from the outputs of elements (7_i−1_), and their second inputs are supplied with a signal from output *B*_2_, i.e. in the case when there is a logical unit at this output, then elements (10_i_) do not affect the operation of the system.

If a logical zero is formed at the specified input, then the second stage de facto cyclically performs the operation

33 $${q}_{i}^{\left(5\right)}={q}_{i+1}{q}_{i}{q}_{i-1}$$

This operation in the case corresponding to Fig. 1b, leads to the appearance of a logical unit at only one of the outputs of the elements (11_i_), the outputs of which are connected to the inputs of the decision device (12).

Thus, the considered part of the circuit provides unambiguous identification of the digital logarithm for cases corresponding to Fig. 1a–f.

Cases Fig. 1g,h correspond to a logical unit generated at the output *B*_1_ of the adder 9. This signal blocks the operation of the first digital differentiation block and transmits it to the second, arranged in a similar way, with the difference that identification is carried out through the use of functions (29) and (30).

Thus, we have shown that the use of an alternating binary representation for elements of the $GF\left(257\right)$ field allow one to realize all the same advantages that occur when Galois fields corresponding to prime Mersenne numbers are used.

The most important type of field of this type is the $GF\left(257\right)$, since it corresponds to the number of levels of the digitized signal, which is often used in practice. For example, the most commonly used analog-to-digital converters involve the use of exactly 256 levels.

The proposed algorithm allows one to reduce the multiplication operation to the addition operation.

As shown, in particular, in^23^, any operations in the Galois field, given, for example, by a truth table, can be reduced to the operations of multiplication and addition. The electronic circuit providing the multiplication operation has been presented above. The scheme of a universal adder on the modulus of an arbitrary integer was presented in our work^43^ on the basis of modernization of the adder scheme, for which we received a patent of Kazakhstan^44^. Thus, the scheme presented above allows to realize, for example, on-board calculators for UAVs operating as part of a group, as well as to solve similar problems.

### Probable generalization and applications of proposed approach

Let us consider the possibilities for generalizing the proposed approach, although at this stage of research this issue is rather of academic interest. In particular, the next quasi-Mersenne number after 257 is the number 2^16^ + 1 = 65,537.

For any Galois field $GF\left({2}^{n}+1\right)$ we have

34 $${2}^{n-1}\equiv -1\left({2}^{n}+1\right)$$

This follows from the fact that

35 $${2}^{n}+1\equiv 0\left({2}^{n}+1\right)$$

Further, all non-zero elements of the field $GF\left({2}^{n}+1\right)$ are roots of the equation

36 $${x}^{{2}^{n}}-1=0,$$

which directly follows from the theory of finite algebraic fields: the number of non-zero elements of the field is one less than their total number, therefore all elements of the field satisfy an equation of degree ${2}^{n}$.

In accordance with the method used above, it is convenient to represent this equation as follows

37 $${x}^{{2}^{n}}-1={\left({x}^{\frac{{2}^{n}}{2n}}\right)}^{2n}-1=0$$

where $N$ is the number of bits in the binary alternating representation of the field $GF\left({2}^{n}+1\right)$.

Formula (37) takes into account the following fact. $n$ bits are required for representation of non-zero elements of the field under consideration in alternating encoding,

38 $$N={\text{log}}_{2}\left({2}^{n}\right)=n$$

Therefore, there are $2n$ field elements, differing from each other by multiplication by a power of 2 in representation under consideration: in order for a quasi-cyclic permutation to lead to the original result, the field element must be multiplied by ${2}^{2n}$ modulo ${2}^{n}+1$. Consequently, the set of non-zero elements of the field under consideration is divided into 2n subsets, each of which contains $\frac{{2}^{n}}{2n}$ elements.

The values of these quantities for the first 4 quasi-Mersenne numbers are presented in Table 4.

**Table 4:** Values ​​of quantities for the first 4 quasi-Mersenne numbers.

$n$	2	4	8	16
${2}^{n}+1$	5	17	257	65,537
$2n$	4	8	16	32
${2}^{n}/2n$	1	2	16	2048

It can be seen that for Galois fields corresponding to the first three quasi-Mersenne numbers, it certainly makes sense to identify subsets whose elements differ by multiplication by a power of 2. Already for the fourth quasi-Mersenne number, the advisability of using this approach is, at a minimum, not obvious. Elements similar to those shown in Fig. 1 becomes not 8, but 1024.

In general, Table 4 shows that the proposed approach is indeed appropriate to apply to specific Galois fields *GF*(17) and *GF*(257), which are of practical interest. Taking into account the results obtained in^36^, the field *GF*(5) may also be of interest as conjugate (in the sense of digital logarithm) with the field $GF\left({2}^{2}\right)$ to simplify the operations of four-valued logic.

Let us show that the results obtained are indeed of interest from the point of view of combining methods of digital signal processing and multi-valued logic for the case when the signal is reduced to 256 discrete levels.

As shown in^23,28^, to reduce the operations of multivalued logic to algebraic ones, it is advisable to use algebraic analogue of the δ-function

39 $${\delta }_{i}\left(x\right)=1-{\left(x-{x}_{i}\right)}^{{p}^{n}-1}$$

where $x$ is the current variable that takes values in the Galois field $GF\left({p}^{n}\right)$, ${x}_{i}$ is the *i*-th element of the field in question.

This function has the following property

40 $${\delta }_{i}\left(x\right)=\left\{\begin{array}{c}1,\,\,\,x={x}_{i}\\ 0,\,\,\, x\ne {x}_{i}\end{array}\right.$$

This property follows from the general theory of Galois fields, according to which, for an arbitrary element of a Galois field containing ${p}^{n}$ elements ${x}^{{p}^{n}-1}=1$.

The use of algebraic analogue of the *δ*-function allows, in particular, to reduce any operations of ${p}^{n}$-logic (logic, the number of values of a variable is equal to ${p}^{n}$) to an explicit algebraic form^23,28^. In relation to the logical function of two variables and the Galois fields *GF(p)*, the corresponding expression has the form

41 $$F\left(x,y\right)=\sum_{i,j=0}^{i,j=p-1}f\left({x}_{i},{y}_{j}\right){\delta }_{i}\left(x\right){\delta }_{j}\left(y\right)$$

where the values $f({x}_{i},{y}_{j})$ form a truth table (recall that the operations of multi-valued logic are currently specified through truth tables^29^).

Relation (41) clearly expresses the well-known fact: if the number of values of variables of multivalued logic is equal to an integer power of a prime number, then logical operations can be reduced to calculations in the conjugate Galois field.

If this condition is not met, then it is advisable to modify the algebraic analogue of the δ-function using the digital logarithm operation^36^. Moreover, it is also advisable to use this approach for the case of the field $GF\left({p}^{n}+1\right)$.

Specifically, we can compose an algebraic analogue of the δ-function in the following form

42 $${\delta }_{{n}_{i}}\left(n\right)=1-{\left({\theta }^{n}-{\theta }^{{n}_{i}}\right)}^{{p}^{n}}$$

In this formula, $n$ and ${n}_{i}$ denote integers that correspond to the elements of the field $GF\left({p}^{n}\right)$. It is assumed that the values of the function itself ${\delta }_{{n}_{i}}\left(n\right)$ belong to the field $GF\left({p}^{n}+1\right)$, on which the mapping is performed; $\theta$—is a primitive element of the field $GF\left({p}^{n}+1\right)$, i.e. such an element which degrees are exhausting all non-zero elements of a given field.

This formula is convenient in that it allows you to get an expression for any operation carried out in terms of 256-valued logic to algebraic ones.

Indeed, using (42), we have

43 $$Q\left(n,m\right)=\sum_{i,j=0}^{i,j=p-2}{Q}_{i,j}{\delta }_{{n}_{i}}\left({\theta }^{n}\right){\delta }_{{m}_{j}}\left({\theta }^{m}\right)$$

where the quantities ${Q}_{i,j}$ re associated with the elements of the truth table ${p}^{n}$-valued logic in the following way

44 $${Q}_{i,j}={\theta }^{{n}_{i,j}}$$

where ${n}_{i,j}$ is the number corresponding to the element of the truth table with numbers $i,j$.

Formula (43) can be considered as a function of a pair of elements of the field $GF\left({p}^{n}\right)$, corresponding to the numbers $n$ and $m$, and taking a value in the field $GF\left({p}^{n}+1\right)$. Formula (43) admits an obvious generalization to a function of an arbitrary number of elements from the field $GF\left({p}^{n}\right)$.

When substituting two arbitrary ${n}_{0}$ and ${m}_{0}$ into formula (43), due to (42), we have

45 $$Q\left({n}_{0},{m}_{0}\right)={Q}_{{n}_{0},{m}_{0}}$$

Only one term in the formula (43) is non-zero.

It can be seen that in order to go back to the elements of the field $GF\left({p}^{n}\right)$ when using formula (43), namely the digital logarithm operation is required, which is proposed in this work for the important special case of $GF\left({2}^{8}+1\right)$.

Therefore, in the future it is possible to develop systems that directly operate in 256-valued logic.

### Algorithms for controlling groups of unmanned vehicles as an area of application of the obtained results

It was noted above that one of the practical applications of calculators operating with Galois fields of relatively small size is the development of algorithms for controlling groups of UAVs, which, we emphasize again, are currently attracting increasing interest of researchers^16,17^. In this section, we will try to demonstrate that for this purpose, the digital differentiation operation realized thanks to the developed approach is essential.

The problem of group control of robots for various purposes has been considered in the literature for a very long time^16,45,46^. This includes vehicles moving in a 3-D environment^17,47^. Various methods are used for its solution, in particular, those based on self-organization (Self-adaptive collective motion) of UAV groups^48^, on machine learning^49^, on the use of graph theory^50^. There are known works that consider a modernized Olfati-Saber algorithm using a virtual leader who is tracked by all UAVs forming a group^51^. In^52^, algorithms built using artificial intelligence combined with IoS have been proposed to control a swarm of UAVs. On this basis, a self-organizing ZigBee network is simulated in the cited work.

However, the decentralized robot control algorithm, which only takes into account information about the positions of other system elements but not about the directions of their movement, have significant limitations^52^. This difficulty is partially overcome in^20^.

The control algorithms for a system of multiple UAVs considered in^53^ also focus on distributed control centered on the so-called leader–follower consensus, which ensures that the entire swarm as a system whole moves according to a predetermined trajectory. In^54^, where the drone swarm is considered from the perspective of Networked Control Systems (NCS), the role of on-board computing systems for controlling the UAV swarm as a systemic whole is emphasized. In^55^, the problem of interfacing an artificial neural network with a UAV swarm was solved, which, among other things, provides for maintaining a given distance between the elements of the swarm, as well as to maintain the formation of the group.

Thus, the solution of the problem of controlling the UAV swarm as an integral system, as follows from the above, is closely related to the solution of the problem of information processing by onboard computing systems.

It can also be seen that this problem can be solved by a variety of means. However, there is an essential nuance, which, in particular, is demonstrated by the results of^54,55^. On the one hand, the amount of information received by the individual elements of the UAV swarm should not be excessively large. On the other hand, it should be sufficient, for example, to allow a particular element of the group to take an adequate position in the swarm (especially when the swarm is ordered).

This returns to the issue of using fuzzy logic to control groups of UAVs, which was considered in particular in^21,22^.

In^21^, an algorithm based on fuzzy logic is proposed that can control a swarm of robots in order to maintain a leader–follower formation without collisions with other agents in the swarm. Simulations have shown that the swarm moves as a unit following the leader. In^22^, algorithms based on fuzzy logic were used to solve the problem of fault tolerance of a group of several autonomous UAVs when they form a formation in the shape of a certain geometric figure. The proposed approach based on fuzzy logic allows on-board control units of each UAV to make their own decision in a decentralized manner. Such decisions, including the possibility of changing the configuration of the whole group.

Consequently, it is reasonable to raise the question of creating computational means for use in on-board computing units of UAVs, which will be purposefully designed to perform operations of odd logic.

As noted above, such operations can be reduced to multi-valued logic operations as demonstrated, for example, in^23^. Further, it is possible to lead these operations to computations in Galois fields or finite algebraic rings^28^. It is this fact that determines the significance of using the operation of digital logarithmization, which can be realized by quite simple means using the proposed approach.

We will show that when we pass to the operations of multivalued or fuzzy logic, the use of Galois fields has very significant advantages compared to the situation when the operations of multivalued or fuzzy logic are represented in tabulated form.

As emphasized, in particular, in^56^, the development of methods for controlling groups of UAVs is inextricably linked to the problems of providing secure communication channels. The main methods of such protection are based on the use of cryptography, but it is also relevant to provide protection at the physical level, for which various approaches can be used, a review of which is given in the cited work. These include, for example,^57,58^.

Among them, one of the methods of physical protection of UAV onboard computers from third-party information influences is the implementation of appropriate algorithms not at the level of programs executed by the onboard computer, but at the level of electronic circuits. Any program remains unguaranteed from third-party interference. On the contrary, if an algorithm is realized at the level of electronic circuitry, it is much more difficult to transform it due to third-party informational influences.

It is this circumstance that determines the advantages of the approach based on the use of explicit algebraic expressions expressing the operations of multivalued logic over their representation in tabular form. Indeed, the use of the tabular form obviously implies the installation of this or that program on the on-board computer. On the contrary, as it was shown in^23,28^ on concrete examples, the representation of operations of multivalued logic in algebraic form allows to realize electronic circuits performing the corresponding computations without using software.

Further, among multivalued logics, as it has been clearly shown, in particular, in^23,28^, a special place is occupied by logics complementary to Galois fields $GF\left({p}^{n}\right)$. In this case, the reduction of operations of multivalued logics to algebraic form turns out to be the simplest. In particular, the algebraic expression to which any operation of such a multivalued logic is reduced contains only the operations of multiplication and addition. Even in the case when the number of values of a multivalued logic variable is only one less than a prime number (or its degree), we have to introduce additional algebraic operations into consideration (the operation of digital logarithmization and its inverse^28^). However, for the purposes of building algorithms for controlling groups of UAVs, this difficulty is not fundamental, since it is always possible to introduce “empty” commands, supplementing the number of commands to a convenient value ${p}^{n}$.

Consequently, the effectiveness of any UAV group control algorithms based on the use of fuzzy or multivalued logic can be evaluated on the basis of their compliance with formulas similar to formula (43), which leads the operations of multivalued logic of the considered type to an algebraic expression (and further—to the realization in the form of a specific electronic circuit).

We emphasize that any algorithm of the considered type can be regarded as a special case of the above formulas, since any operation of multivalued logic of the considered type is reduced to an expression of this type.

One cannot but see that from the point of view of realization in the form of an electronic circuit the most resource-intensive operation is the operation of raising in degree, on which the algebraic delta-function (42) is built.

The corresponding calculations can be simplified by using the digital logarithm operation, which is performed by the circuit shown in Fig. 2. In this case, the operation of increasing in degree is reduced to a multiplication operation. Moreover, due to the specificity of the considered field $GF\left(257\right)$ the digital logarithm operation maps non-zero elements of this field to elements of the field $GF\left({2}^{8}\right)$, computations in which are realizable on the basis of standard elements corresponding to binary logic.

There is every reason to believe that control algorithms of UAV groups in the foreseeable future will be oriented to some standards similar to those currently developed in the field of digital signal processing, television, etc. Proceeding from the fact that such algorithms, in the end, conveniently lead to calculations in Galois fields (and their realization through electronic circuits), it is acceptable to assume that the expected standard will be associated with a specific Galois field.

Taking into account that the most resource-intensive is the operation of raising to degree, it seems reasonable to focus on those Galois fields which, on the one hand, have enough elements to cover the needs of practice, and, on the other hand, allow to realize the operation of digital logarithmization by the simplest means possible.

It is this criterion that the field $GF\left(257\right)$ considered in this paper satisfies, which is complementary (from the point of view of performing the operation of digital logarithmization) to the field $GF\left({2}^{8}\right)$, the computations in which can be realized on the basis of standard elements.

It is also appropriate to note that the circuit providing digital logarithmization is also realizable on the basis of type elements corresponding to binary logic. This, among other things, means that to realize the proposed approach in practice it is possible to use microcircuits with programmable logic structure, which are being actively developed at present^59^. Let us also note that there is a possibility to realize an adder on the modulus of an integer with adjustable modulus value, which is also built on typical logic elements^43^. Thus, there is a possibility for realization of on-board UAV calculators, completely based on calculations in Galois fields.

Note also that electronic circuits providing computations modulo integer (adders and multipliers) have been actively developed recently^60,61^. Such devices, obviously, can be used also for computations in Galois fields. Among others, there are known constructions of modulo ${2}^{n}+1$ calculators that satisfy fields of the considered type^62,63^. Investigations in the field of modular adders and multipliers are also reflected in the patent literature^64,65^. The disadvantage of existing modulo adder schemes, however, remains their complexity. For example, the adder scheme presented in cited reports can be replaced by a substantially simpler one^44^. A similar conclusion is true for the schemes proposed in^62–66^. The operation of digital logarithmization, based on the algorithm proposed in this paper and realized in the form of the scheme of Fig. 2, allows to pass from the multiplication operation to the addition operation, and the latter is performed on the basis of schemes corresponding to the usual binary logic, as it was shown above.

## Conclusion

Thus, the use of finite algebraic structures is of interest not only for the purposes of cryptography, where the use of algebraic fields or algebraic rings of large size is required. Of no less interest are problems in which the number of elements of algebraic structures remains relatively small, which, in particular, is demonstrated by the example of algorithms of UAV flight computers operating as part of a group.

The fact, as well as results obtained once again show that for applied using it is extremely important to take into account the specifics of concrete Galois fields. In particular, this applies to the field $GF(257)$, which corresponds to one of the quasi-Mersenne primes, i.e. numbers that can be represented in the form $p={2}^{n}+1$.

This field is associated with the number 256, which corresponds to one of the most important standards used in modern digital technologies.

For numbers corresponding to fields $GF({2}^{n}+1)$, it is convenient to use an alternating encoding, in which multiplication by the number 2 modulo $p={2}^{n}+1$ corresponds to a quasi-cyclic permutation of binary symbols, i.e. cyclic permutation with a change in the sign of the permuted element.

This encoding allows one to implement a simple digital logarithm algorithm, which allows one to reduce the multiplication operation to the addition operation, etc.

Specifically, for the $GF(257)$ field, the digital logarithm operation is simplified due to the fact that the set of non-zero elements of this field can be divided into 16 subsets, the elements of which differ from each other by a quasi-cyclic permutation of binary symbols. As a result, the operation of digital logarithm for a given field leads to the identification of an element by belonging to one of these subsets.

It is important that the operation of digital logarithm in the field under consideration, which leads the multiplication operation to the addition operation, can also be implemented using relatively simple electronic circuits. A corresponding example is presented in this work. This scheme, along with the scheme of adder modulo integer with adjustable modulus value, proposed in^43^, allows to realize any operations in the field GF(257), for example, set through the truth table. In the future, this approach can be the basis, for example, for on-board UAV calculators acting as part of a group. Operations in Galois fields of relatively small size are also of interest in the future for the development of new artificial intelligence systems, approaching the biological prototype by the principle of operation, the functioning of which cannot be reduced to binary logic.

## Data Availability

All data generated or analyzed during this study are included in this published article.
